# An Exploratory Study Into the Negotiation of Cyber-Security Within the Family Home

**DOI:** 10.3389/fpsyg.2020.00424

**Published:** 2020-03-12

**Authors:** Kate Muir, Adam Joinson

**Affiliations:** Applied Digital Behaviour Lab, School of Management, University of Bath, Bath, United Kingdom

**Keywords:** cyber-security, security, technology, family, communication, negotiation, qualitative interviews

## Abstract

Given the increasingly young age that children are using technology and accessing the internet and its associated risks, it is important we understand how families manage and negotiate cyber-security within the home. We conducted an exploratory qualitative study with thirteen families (14 parents and 19 children) in the south-west of the United Kingdom about their main cyber-security concerns and management strategies. Thematic analysis of the results revealed that families were concerned about cyberbullying, online stranger danger, privacy, content, financial scams, and technical threats. Both parents and children drew on family, friends and trusted others as resources, and used a variety of strategies to manage these threats including rules and boundaries around technology, using protective functions of technology, communication and education around safety. There were tensions between parents and children over boundaries, potentially putting families at risk if children break household rules around cyber-security. Finally, parents expressed the feeling they were in a ‘whole new world’ of cyber-security threats, and that positive and negative aspects of technology must be constantly balanced. However, parents also felt that the challenges in managing family security are the same ones that have always faced parents – it is just that the context is now digital as well as physical.

## Introduction

Children start using digital technologies at a young age: in 2013, 75% of American children under the age of nine used a tablet or smartphone in the home (Common Sense Media, 2013) and by 2018, 42% of 5–7 year olds and 47% of 8–11 year olds in the United Kingdom owned their own personal tablet (Ofcom, 2019). Further, despite the age limits of most popular social networking sites being 13 upwards, in 2018 12% of 9-year olds in the United Kingdom reported using one or more social media sites, rising to 34% by age 11 (Ofcom, 2019). There are dangers involved in being online at a young age: approximately 16% of 8–11 year olds and 31% of 12–15 year olds in the United Kingdom have reported seeing something inappropriate online (Ofcom, 2019) and worryingly, 22% of 12–15 year olds in the United Kingdom report being contacted by a stranger online (Ofcom, 2019).

Evidence suggests that children have some limited perceptions of the dangers of going online. For instance, Zhang Kennedy et al. (2016) reported that children aged seven to eleven articulated concerns about privacy threats, seeing inappropriate content online (such as swearing) and online strangers. Other research concurs that young children (between the ages of 10 and 12) show awareness of both the risks and negative aspects of going online (such as cyber bullying) and the importance of cyber-security, including controlling and being aware of one’s digital footprint (Buchanan et al., 2017; Murray and Buchanan, 2018). In contrast, young children can also report unfamiliarity with the concept of the internet, implying low levels of awareness of its associated dangers (Edwards et al., 2018). Thus, children’s awareness of cyber security and online dangers is uneven, with differences amongst children according to age, gender and socioeconomic status (Livingstone et al., 2017). Further, it is unclear to what extent children’s awareness of online risks translates into secure behaviors (UK Government, 2017), meaning further research is necessary to understand to what extent children are aware of the dangers of cyber-security threats, and their level of knowledge as to how to address them.

Within the home, we might assume that parents are naturally in charge of cyber-security. After all, parents guide their children toward the most age-appropriate television programs to watch and video games to play (Nikken and Schols, 2015; Schaan and Melzer, 2015), and mediate their children’s use of the internet (Livingstone and Helsper, 2008). Parenting styles, in terms of patterns or combinations of parenting practices (Darling and Steinberg, 1993) may influence the type of approach adopted by parents in their management of household cyber-security. Baumrind (1971, 1991) described an influential typology of parenting styles in which parenting practices differ along dimensions of support (the affective dimension of the parent-child relationship, including warmth and involvement) psychological and behavioral control (controlling, shaping and regulating children’s behavior). In this typology, parenting styles are defined as authoritarian (high control but low involvement) authoritative (high control and high involvement), indulgent or permissive (low control but high involvement) and neglectful (low control and low involvement). These parenting styles have been supported both conceptually and empirically in relation to developmental outcomes for children (see Kuppens and Ceulemans, 2019, for a recent review). General parenting style then, may dictate the type of approach parents take to managing cyber security in the family.

However, there is an argument that rather than parents being solely in charge of household cyber-security, *children* are becoming the leading experts in technology use in the household (Livingstone, 2009). Indeed, children influence their parents, particularly in encouraging them to take up social media and teaching them how to use new technology in the home such as computers and the internet (De Mol et al., 2013; Correa, 2014; Correa et al., 2015). So, if children influence their parents in their use of new technology, how does this relate to the management of cyber-security within the home? We propose that instead of parents (or indeed, children) being *solely* in charge, perhaps the management of cyber-security in the family home is *negotiated between* parents and their children (Agosto and Abbas, 2017). Prior research has examined such processes of negotiation in the context of technology use in the family home. For instance, there is evidence for conflict in parent-child relationships when parents try to restrict children’s use of technology like tablets (Blum-Ross and Livingstone, 2016; Hadlington et al., 2019). This conflict is even more evident when children take the lead in technology use in the home, as traditional power roles of parents versus their children are reversed (Holloway and Valentine, 2002; Nelisson and Van den Bulck, 2018). However, it is yet unclear if similar processes apply to the management of cyber security within the home. It is possible, yet unexplored, that tensions could also characterize the negotiation of cyber-security within the family home. It is important that we understand this issue, as if there are tensions between parents and children in the management of cyber-security, this could leave the whole household potentially vulnerable to cyber-attacks. Pertinently, the United Kingdom Government has recently made understanding how families manage cyber-security a priority, showing there is a research gap in this area (UK Government, 2017).

Thus, we aim to increase our understanding of what parents and children in the United Kingdom perceive as cyber-security threats, and how such threats are managed and negotiated within the family home. Given this is an exploratory area, we take a qualitative approach to gather initial indications as to the types of cyber-security threats that families in the United Kingdom are concerned about, and the negotiating processes within the family home in terms of managing these threats. Our initial findings will guide further larger scale examinations of the processes and factors involved in family negotiation in cyber security and inform knowledge as to how best to support families in their cyber-security management.

We use the Model of Social Change and Human Development developed by Pinquart and Silbereisen (2004) to frame our investigation into the negotiation of cyber-security in families. The model proposes that the effects of any social change upon individuals are mediated through microsystems, or immediate personal contexts such as the workplace, school and family. Wide scale social changes result in changes to these personal contexts, placing demands on the individual. As an example, a wide scale economic crisis (*the social change*) may result in job losses in the workplace (*the microsystem*), placing financial demands (*the localized demand*) upon an individual. The model predicts that how individuals deal with these new demands, such as trying new options or avoiding risks (*their coping response*) will depend on several factors. These include the *resources* the individuals have available to them, such as social support or individual knowledge, and the factors that limit their ability to cope with new demands, such as economic or social *vulnerabilities*. Thus, individuals vary in the resources they can draw upon to deal with changes in their microsystems, and individuals with relatively more rich sources of support are more likely to have a variety of coping responses that enable them to adapt to social changes, both socially and psychologically (*their individual outcomes*).

**FIGURE 1:**
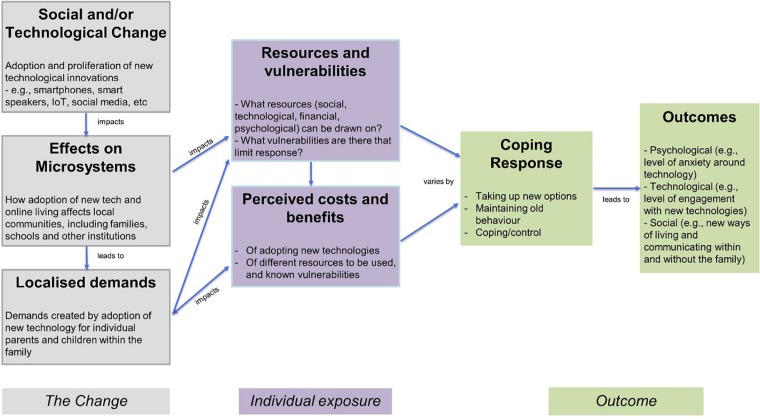
Behavioral model of social change and human development by Pinquart and Silbereisen (2004), adapted to highlight social change in terms of technological development.

In this study we apply the model to one particular social change, the introduction and proliferation of new technology. We use the framework provided by the model to explore how this social change impacts upon the management of security within the microsystem of the family home. Figure 1 (above) presents the Pinquart and Silbereisen (2004) model, adopted to highlight the proliferation of new technology in society as the social change of interest (such as the introduction and rapid adoption of smartphones, the popularity of social media and the rise of Internet of Things (IoT) technology).

We use this model as a framework to structure our investigation into the ways in which cyber-security is perceived and managed in families. The model predicts that the effects of new technology being introduced into society (*the social change*) upon individual parents and children are mediated through their use in the family home (*the microsystem).* New technology being used in the family home places extra *demands* upon individual parents and their children, in terms of managing the security implications of these new technologies. According to the model, how members of individual families respond to these demands in terms of their *coping responses* (i.e., how they manage cyber-security within the home) is influenced by the *resources* they have available to them, and any perceived *vulnerabilities*. These resources could take the form of individual level knowledge about cyber-security or a social resource they can access, such as a friend or workplace IT professional who could provide advice. The model also predicts that families evaluate the *costs* and *benefits* of adopting innovations in technology in terms of how much technology poses a risk to family security. Together, a family’s resources, perceived vulnerabilities and perceptions of costs and benefits determine *coping responses* in terms of strategies families use to manage their security concerns. In turn, these predict the *psychological, technological, and social* outcomes of technology adoptions at the individual and society level.

Given the exploratory nature of our study, we took a qualitative approach to explore this framework in relation to how cyber security is perceived and negotiated within families in the United Kingdom. We conducted qualitative interviews with families with children between the ages of six and sixteen in the south west of the United Kingdom, to explore their views and experiences of managing cyber-security within the family home. We thus draw upon this framework to structure our research questions, design the topics covered within the qualitative interviews and to guide thematic analysis of participants responses. Based on the elements in the model as described above and in Figure 1, we formed the following research questions to guide our qualitative investigation:

**RQ1**: What are the *demands* upon families in terms of the cyber-security risks they are concerned about?

**RQ2:** What *resources* do families draw upon to manage these demands, and what *vulnerabilities* limit their response?

**RQ3:** What are the *costs* and *benefits* of using new technology for families?

**RQ4**: What are the *coping responses* that families use in terms of strategies to cope with the demands?

**RQ5:** What are some of the *social, technological and psychological outcomes* of the changes in technology adoption for families in the United Kingdom?

## Materials and Methods

We advertised the study via local parenting groups and participant recruitment websites, to families in the south west of the United Kingdom who had children between the ages of 6 and 16 year olds living within the home, resulting in thirteen families volunteering to take part. We conducted face-to-face semi-structured interviews with these thirteen families, with topics centered around our five research questions. We explored the *demands* faced by families in terms of their main cyber-security concerns, what *resources* they draw on and *vulnerabilities* they perceive, what families perceive to be the *costs* and *benefits* of using technology in the home, the *coping strategies* they use to manage the demands, and what the *social*, *technological* and *psychological* outcomes are of managing these demands. Data was collected between October 2018 and April 2019.

### Ethical Considerations

Ethical approval for the study was granted by the Social Science Research Ethics Committee of the University of Bath, United Kingdom (ref S18-013). Upon responding to the study advertisement and again before the start of the interview, parents were provided with an information sheet and consent form which detailed the aims of the study and the topics that would be covered in the interviews. Children were provided with a child friendly version of this study information sheet and consent form. After ensuring that both parents and children had read and understood the information sheet, parents provided written consent to take part, and consent for their children to take part. Children provided written assent to take part by writing their name on the child-friendly consent form.

### Participants

We report the characteristics of the thirteen families who took part in the study in Table 1 (below) including age and gender of the adults and children who took part in the interviews or were part of the family but did not take part in the interviews directly. We report the technology present and frequently used in the home, and the most frequently reported activities (on a daily basis) by one or more family members. Fourteen parents took part in the interviews (twelve female, two male) between the ages of 31 and 55 years old (*M* = 43.90, *S.D*. = 6.30) and nineteen children (fifteen males, four females) between the ages of six and sixteen (*M* = 9.63, *S.D*. = 3.09). Note, we use pseudonyms to preserve the anonymity of the adults and children who took part in the study.

**TABLE 1:** Details of study participants, technology ownership and use in the home.

Family	Adults (gender, age)	Children (gender, age)	Devices in the home	Frequent activities
1	Katherine (F, 37) Jack (M, 38)^1^*	Harrison (M, 7)* Aurelia (F, 1)	Computer Fitness wearable Games console Smart thermostat Smart TV Smartphone Tablet	Email Internet Social Media Tasks Work
2	Teresa (F, 42)^1^* Jim (M, 47)	Alfie (M, 8)* Jack (M, 6)*	Computer Fitness wearable Games console Smart TV Smartphone Tablet	Banking Chat Email Internet Shopping Social media Work
3	Daisy (F, 46)^1^* Joseph (M, 52)	William (M, 14)* George (M, 6)*	Computer Games console Media player Smart speaker Smart TV Smartphone Tablet	Chat Email Internet Online gaming Social media Videos Work
4	Julie (F, 43)^1^* Malcolm (M, 49)	Adam (M, 13)* Lottie (F, 12)* Paul (M, 6)*	Computer Fitness wearable Games console Smart lights Smart plugs Smart speaker Smart TV Smartphone Tablet	Banking Chat Email Internet Online gaming Shopping Social media Tasks Videos Work
5	Polly (F, 31)^2^* Charles (M, 55)	Jacob (M, 14) Anna (F, 9)* Darren (M, 7)* Noah (M, 5)	Computer Fitness wearable Games console Smartphone Smart TV Tablet	Banking Chat Email Internet Shopping Social media Videos Work
6	Beth (F, 43)^3^*	Teddy (M, 8) Finley (M, 6) Reggie (M, 5)		
7	Sarah (F, 41)^1^* Bob (M, 45)	Henry (M, 8)* Laurie (M, 6)	Computer eReader Smart speaker Smartphone SmartTV Tablet	Banking Chat Email Internet
8	Alison (F, 46)^2^*	Thomas (M, 12)*	eReader Fitness wearable Games console Smartphone Smartplug SmartTV Tablet	Banking Chat Email Internet Online gaming Shopping Social media Tasks Videos Work
9	Olive (F, 40)^1^* John (M, 37)*	Alfie (M, 7)* Elsie (F, 3)	Computer eReader Smart TV Smartphone Tablet	Banking Email Internet Shopping Social media Tasks Videos Work
10	Jennifer (F, 37)^1^*	Leo (M, 13)* Charlotte (F, 7)*	Computer eReader Fitness wearable Games console Smart Speaker Smart thermostat Smartphone SmartTV	Banking Chat Email Online gaming Social media Videos
11	Cath (F, 47)^3^* Stephen (M, 54)	Lewis (M, 16) Ben (M, 13) Sophia (F, 8) Jude (M, 3)	Computer Games console Smart thermostat Smartphone SmartTV Tablet	Chat Email Internet Online gaming Shopping Social media Tasks Work
12	Samantha (F, 49)^1^*	Charlie (M, 16)* Sean (M, 13)*	Computer eReader Games console Smartphone SmartTV Tablet	Chat Email Internet Social Media Videos Work
13	Rachel (F, 38)^2^* David (M, 46)	Tilly (F, 11)* Freddie (M, 8)*	Computer eReader Fitness wearable Games console Media player Smartphone Tablet	Chat Email Internet Online gaming Social Media Videos Work

*Participated in interview ^1^Parents interviewed separately to children ^2^Parents and children interviewed together ^3^Only parents interviewed. Example devices: Computer (stand-alone PC, Mac or laptop); eReader (Kindle); Fitness wearable (Fitbit); Games console (PlayStation, Xbox); Media player (media streaming devices that plug into digital televisions to play videos from streaming sites like Netflix, such as Chromecast); Smartphone (iPhone, Samsung Galaxy); Tablet (iPad, LeapPad); SmartTV (television that connects to the internet); Smart Thermostat (Nest); Smart Speaker (home voice activated devices, such as Amazon Echo); SmartPlug (Plugs that can be controlled via an app on a Smartphone); SmartLights (Lights can be controlled via an app on a Smartphone, such as Philips Hue).

### Interview Procedure

Twenty-one interviews took place in total. In most cases, one or both parents were interviewed first, separately to their children (*N* = 8), followed by an interview with the children (*N* = 8). During the child interviews, parents remained in the room or nearby in the house to maintain comfort for children. The interviewer was flexible around the family schedule and wishes of the participants: some children declined to participate and in these cases only parents were interviewed (*N* = 2, families 11 and 6) and in other cases parents and children’s interviews were combined as per the family’s wishes (*N* = 3, families 5, 8, and 13). Similarly, although most interviews were carried out in the family home, some interviews took place in a suitable public space according to the wishes of the participants. Parent interviews lasted approximately 1 h whilst child interviews generally lasted 35 – 45 min according to the comfort level of the child.

The interviews were semi-structured in nature, with the use of a general topic guide but being sensitive to the topics that parents and children were interested in discussing within the area of technology use and concerns around cyber-security. Interviews began with a general discussion of technology in the home, what devices were used by family members and how frequently, and how this is managed. We report these details in Table 1. When reporting devices present in the home, participants tended to use the brand name (e.g., iPhone rather than smartphone). Here we report the type of device in order to compare across families that use different brands of the same technology (see Table note for examples of each device).

The general topic guide was created to address our five research questions, with topics centering around the elements within the theoretical framework guiding our study (Pinquart and Silbereisen, 2004). Thus, during interviews with parents, the interviewer introduced discussions around what cyber-security threats family members were concerned about and why (*localized demands*), what (if any) rules and guidelines were in the household around device and internet use, and how they approached cyber-security in the home (*coping strategies*). Discussions also included what social, individual and community *resources* parents drew upon to manage cyber-security, and what families perceived to limit their ability to manage cyber security in the home (*their vulnerabilities*). The topic guide also included discussions around perceived positives and negatives aspects of using technology (*costs* and *benefits*). Additionally, the interviewer discussed how parents felt about managing cyber-security in the home (*psychological and social outcomes*). Children’s interviews covered similar topics in a child-friendly manner, depending on the age of the child. Images of various devices (e.g., tablets, smartphones) were used as prompts for children to discuss how they used technology in the home, and the things they liked and disliked about using the technology, and any strategies they used to limit any concerning aspects of using technology.

At the end of the interview, families were debriefed and given a shopping voucher as a thank you for participating. All interviews were audio recorded using a Dictaphone. The first author conducted all interviews.

### Thematic Analysis

The transcripts were transcribed verbatim by the first author who then coded all the transcripts. We used thematic analysis as our method of qualitative analysis, which is a method for “identifying, analyzing and reporting patterns within data” (Braun and Clarke, 2006, p. 79). We chose to use thematic analysis for its flexibility and ability to provide rich and detailed description of the patterns within our data. The analysis proceeded following the steps outlined in Braun and Clarke (2006). Transcripts were firstly read and re-read to become familiar with the data before initial code generation, capturing general repeated themes within the interviews. These themes were iteratively reviewed and revised. The first author conducted three rounds of coding before a final set of themes and sub-themes were generated, which were grouped around our five research questions relating to the demands faced by families in terms of cyber security threats (RQ1), resources drawn upon by families and perceived limitations (RQ2), the main benefits and costs to using technology in families (RQ3), coping responses utilized by families (RQ4) and finally wider themes around the social, technological and psychological outcomes of using technology in the family (RQ5). All analysis was performed using Nvivo software. An initial codebook was created by the first author based on the final set of themes and sub-themes which was reviewed by the second author along with a random selection of 20% of the transcripts. This led to discussion and clarification on themes and one new sub-theme being generated, and revision of the codebook. The first author then coded all transcripts using the updated codebook. Finally, a random selection of 30% of the remaining transcripts were then reviewed and coded by the second author using the updated codebook. Inter-coder reliability of all final major themes was acceptable with Cohen’s kappa of.68 (95% CI 0.62,0.75) with all disagreements resolved through discussion.

## Results

Table 2 presents a summary of the themes and sub-themes generated in the interviews, along with their prevalence (number of parents and children who mentioned each theme within the interviews). Below, we describe each theme in terms of how they address our research questions. Each theme is illustrated with supporting quotes from participants. We refer to our participants using a pseudonym, followed by their gender and age (e.g., Bob, M, 9 years).

**TABLE 2:** Themes and sub-themes generated in the interviews.

Theme	Subthemes	Prevalence
Demands (Cyber-security threats)	Cyberbullying Stranger Danger Online privacy Online content Financial threats Technical threats	6/14 parents; 6/19 children 6/14 parents; 5/19 children 6/14 parents; 3/19 children 5/14 parents; 3/19 children 7/14 parents; 4/19 children 7/14 parents; 1/19 children
Resources	Family, friends and trusted others Technological solutions	7/14 parents; 8/19 children 5/14 parents; 6/19 children
Vulnerabilities	Limitations in knowledge Insecure settings	6/14 parents 2/14 parents; 4/19 children
Benefits of using technology	Connection and Security	4/14 parents; 4/19 children
Costs of using technology	Behavioral changes	8/14 parents; 2/19 children
Coping strategies	Boundaries Battling the boundaries Monitoring Communication	9/14 parents 6/14 parents 6/14 parents 6/14 parents
Social, Technological and Psychological Outcomes	Deferring and avoiding technology A whole new world Same same but different	4/14 parents 6/14 parents 8/14 parents

### RQ1: What Are the Demands Faced by Families, in Terms of Cyber-Security Threats?

In this section we describe the cyber-security threats that families discussed in the interviews: *cyberbullying*, *stranger danger* (catfishing and other related threats such as grooming), *online privacy*, *online content*, *financial threats* (identity theft, phishing, scam calls, and other threats to family finances) and *technical threats* (hacking and viruses).

#### Cyberbullying

Six parents and six children expressed concerns about the negative impact upon emotional wellbeing caused by cyberbullying. This bullying took several forms, from comments made on social media to being harassed through group messaging apps. Lottie (F, 12 years) talked about how she was careful what she posted on her social media accounts as “*there are people out there who are not very nice and you could get hurt.*” Cyberbullying, over and above more physical forms of bullying, was perceived to have the potential for negative impact as it is ‘always on,’ meaning its impact can be long-lasting and severe, as described by this mother of 8 and 6 years old boys: “*Its high because it can have a big detrimental effect on kids. The traditional bullying in the playground is almost easier to deal with if people are being pushed around it’s a physical thing that people can see, and can understand but cyber bullying can happen in so many different ways and is very subtle and really influential to how someone is feeling*” (Sarah, F, 41 years). Some children echoed this feeling, acknowledging how the ‘always on’ nature of digital communication contributes to cyber-bullying, as highlighted by this 14 years old boy: “*the thing with the cyberbullying, with the notifications, one night I was up until like 10.30pm, up with the phone bleeping and I had to keep looking at it*” (William, M, 14 years).

#### Stranger Danger

Six parents discussed how their security concerns were based on the physical and emotional ramifications of their children interacting with strangers online and forming relationships with people that they had not actually met in person. The media was quoted as a source of information about stranger danger, as in this narrative from Beth (F, 43 years) “*It’s the stranger danger, purely, aware of – reading on the news about a teenager being murdered, met someone from an online game.*” Concerns around online ‘stranger danger’ covered a range of issues, such as catfishing, online grooming and cyber stalking. As Alison (F, 46 years) explained, “*We are all very aware that it is real that children are groomed via the internet for exploitation.*” This awareness or concern of the dangers of strangers on the internet was echoed by some of the children (5/19 children), as explained by this 13 years old boy when discussing forming relationships online (“*me and mum were talking about how dangerous it could be, and there are people who aren’t that nice or good*”: Sean, M, 13 years). Younger children in our study also showed awareness of online stranger danger, as described by this 9-year old girl, “*Is this catfish, I know about this. Its where someone like*…*say I’d said I was called Lilly, but then I actually go and meet them and*… *I’m not Lilly*…*I think that’s mean to other people*” (Anna, F, 9 years).

#### Online Privacy

Privacy loss, in terms of losing privacy due to posting personal information online, was raised by six parents as a concern. However, four of these parents saw this loss of privacy as an inevitable part of digital life and not in itself constituting a cyber-security concern. As this parent stated, she didn’t think that the content she posted online posed any security risks: “*This for me, is medium to small – if I put something on social media, good luck to you, I don’t put much. If I put a photo, you are welcome, that’s the sort of thing. So privacy, not a problem*” (Julie, F, 43 years). Contrarily, these same parents often discussed privacy settings on social media as being an important aspect of family cyber-security management: “*all their settings are all on private, they are not allowed any public Instagram or whatever, not allowed to be public, only private settings*” (Julie, F, 43 years). Thus, there seemed to be a disconnect in the minds of some parents in our study between losing a sense of privacy by using social media, but this *not* being a security concern, and at the same time using privacy settings as a way of protecting family members online. Some children also raised online privacy as a concern (3/19 children) and these children showed an understanding that posting information online (on social media, for example) could lead not only to a loss of privacy but could also constitute a security risk, as articulated by this 9 years old girl: “*then also don’t put your like where you live, your school, your address, all your information online because it, then bullies and stuff can come over to your house and start killing you and stuff*.” (Anna, F, 9 years).

#### Online Content

Concerns about children encountering inappropriate content were mostly identified as a concern by parents of younger children (one parent of a 13 years old boy and four parents of children younger than 8 years). One example was concerns around their children hearing inappropriate language (“*every now and again he will find something, he will say ‘oh Mummy I’m sorry I heard a swear word’ because he’s clicked on something which he shouldn’t have done*”: Daisy, F, 46 years, speaking about George, M, 6 years). Younger children (3/19 children) also expressed awareness of the potential for the internet to show them things they didn’t want to see, as observed by Darren, M, age 7: “*Google, it can show you bad stuff*…*it could show you really mean stuff or rude stuff*.”

#### Financial Scams

Included in this category are security threats which impact on the finances of the family, such as identity theft, phishing scams and scam calls, raised by seven parents and four children. Interestingly, parents who discussed financial threats described them as an inconvenience which lacked the ability to seriously impact on family life. Sarah (F, 41 years) said this about an experience of identity theft: “…*it’s a bit annoying and you feel a bit upset but it didn’t impact us massively because we got the money back*.” These parents felt that because they had experience in spotting scam emails or other scams that the threat level was low, because they knew how to deal with them: “*If its linked to like financial, I don’t really think – I’m quite savvy about those so I don’t see that as a big threat to me, because I can spot them quite quickly*.” (Samantha, F, 49 years). In contrast, four children expressed their concerns about the financial implications of identity theft; in their eyes, this means a loss of your financial security with potentially severe consequences. Charlie (M, 16 years) commented “*because as soon as someone has your credit card details, they can pay for things, log into your bank, and that’s it you are basically screwed.”*

#### Technical Threats

Seven parents highlighted that they were aware of ‘technical’ threats – those which concerned attacks on their devices which could result in data loss or loss of functionality, such as viruses. However, these parents were generally less concerned about such threats, referring to a trust in the technology itself to keep them protected from such things, “*I mean ultimately they can be horrible [referring to viruses] that can do horrible things to your laptops and devices, but you know Apple is a fairly closed system and I trust that*” (Olive, F, 40 years). Children did not raise any technical threats as a concern, apart from one 12 years old boy who discussed the threat posed by phone hacking, based on a prior negative experience: *“*…*they hacked my phone and they were sending very rude messages*” (Thomas, M, 12 years).

### RQ2: What Resources Do Families Draw on to Manage These Threats, and What Are Their Perceived Vulnerabilities?

Families had a range of resources that they drew on to manage the cyber-security threats posed to their household. We clustered these resources into two sub-themes of *family, friends and trusted others* and *technological solutions*. In terms of vulnerabilities, parents identified their own *limitations in cyber-security knowledge* and both parents and children discussed *insecure default settings* on social media or websites.

#### Family, Friends, and Trusted Others

Seven parents identified friends, family, and trusted others as a valuable resource. When they ran up against a problem with technology in the home – be it functional or cyber-security related – their first port of call was asking others with perceived greater technological know-how for help, whether that be their friends, family, colleagues, or a trusted brand: “*I’m going to O2, because I’m thinking to myself I need to tighten down on their security for their sake but not knowing how to do it, so that’s my biggest challenge, so I need outside help basically*” (Daisy, F, 46 years). Children (8/19 children) described turning to adults for help if they encountered any threats or concerns online: “*if it comes up with bad screen just go tell your adult and they will sort it out*” (Darren, M, 7 years). Children also relied on *emotional* guidance from their parents, including how to navigate friendships conducted online and how to avoid receiving negative comments on social media posts. Some parents (7/14 parents) relayed stories of providing emotional support to their children who were the victims of cyber bullying. So, parents provided not only technical support to younger children, but had an important role in supporting their children in dealing with the emotional fallout of their usage of new communication technologies.

#### Technological Solutions

Some families (5/14 parents, 6/19 children) described technological ways of protecting the family home, some of these more technical than others, depending on their level of expertise. This parent, for example, had a background in IT which he utilized to keep his family safe: “*I do stick to a broadband provider that provides a number of things, I specifically request the floating IP address*… *that means it’s very difficult to actually find where our communications are coming from, I ask for a broadband provider that has a built-in firewall with additional firewalls that we have on our equipment.”* (Jack, M, 38 years). The technological solutions used by families included installing antivirus software on their devices such as McAfee (described by Adam, M, 13 years) or using devices specifically designed for children such as the LeapPad (described by Olive, F, 40 years). Parents also chose to use parental settings or limits on devices as a technological way (as opposed to physically monitoring) of ensuring age-appropriate content (*“all their tablets*…*I set them all so they can only go age appropriate apps and websites*”: Polly, F, 31 years). Children were also aware of some ways in which they could utilize the protective qualities of the technology itself, including child-friendly search engines (such as YouTube Kids, described by Anna, F, 9 years). Some children described using the Hector’s World Safety Button in school, which is a child-activated piece of software that children can use if they see something upsetting on screen (“*if there is something scary that you don’t like, you press on hector the dolphin*”: Henry, M, 8 years). Using passcode locks on phones and devices was also mentioned by children as a safety measure (described by Harrison, M, 7 years and Charlotte, F, 7 years). One child was aware of keeping their device up to date as a way of protecting their game data *(“Lucky that I updated it because people can’t actually make data loss happen because I pressed update on the Apple iPad”:* Harrison, M, 7 years).

#### Limitations in Cyber-Security Knowledge

Six parents expressed the feeling that their technological knowledge was limited, and this impacted their perceived ability to protect their children from encountering threats online, instead having to rely on help from others (“*The safety side of it, you can’t keep them safe if you can’t understand something”:* Jennifer, F, 37 years). Some parents felt that the speed in which technology was evolving made it difficult for them to keep on top of the security implications: *“My kids talk to me a lot about different stuff and I still don’t think I can scratch the surface of everything that is out there. And so how can I possibly keep up and assess the risk of everything?*” (Samantha, F, 49 years).

#### Insecure Default Settings

Some families (2/14 parents, 4/19 children) also highlighted the difficulty of maintaining secure online practices when settings on social media and devices can leave them vulnerable to security and privacy violations unless explicitly changed. This 13 years old boy discussed his difficulties in changing the default privacy settings on Instagram: “…*its default public. That’s really bad I think they should change that, I don’t know about Facebook and Twitter but on Instagram it was quite hard to find which surprised me*” (Sean, M, 13 years). Similarly, one family (Family 13) discussed how they felt forced into installing a particular piece of software on their gaming platform, which left them feeling vulnerable to privacy violations: *“When we bought him x box live because a lot of the games need it now, we discovered with the connect joined up as well we could hear what was happening in people’s rooms. There is a microphone and you can hear, if they have obviously the same sort of thing, you can hear what is going on in the background*” (Rachel, F, 38 years). Thus, some families in our study expressed the feeling that their ability to control household cyber security was limited because of properties within the technology itself.

### RQ3: What Are the *Costs* and *Benefits* of Using New Technology for Families?

Both parents and children discussed how the benefits and costs of using technology were constantly balanced. Some parents (4/14 parents) acknowledged the benefits of innovations in modern technology such as the internet, in terms of easy access to knowledge (“*it’s not that the internet is bad, obviously I use it all the time myself and I’ve learned so much, that I can’t even think what life would be like without it*”: Cath, F, 47 years) and staying connected as a family (*“I must admit that’s the way of the world, but I do feel safer with my daughter especially having one. It makes my life easier with both of them having mobiles*.”: Julie, F, 43 years). Some children (4/19 children) were also perceptive in the advantages of using technology particularly for communicating with their peers “*It’s a good way of keeping in contact and its really easy and quick. So that’s mainly what I use my phone for*.” (Charlie, M, 16 years).

The positive aspects of using technology were balanced out by its downsides, which mainly consisted of the effects that technology can have on behavior. Charlie (M, 16 years) and Sean (M, 13 years) both expressed concerns that they were becoming addicted to their phones, and they were working to actively cut down their phone use (“*you can just get really addicted, you just can’t get off it. It becomes like a habit, so like once you do something your thought goes to your phone. I don’t think that’s good*.” Charlie, M, 16 years). This concern was echoed amongst parents (8/14 parents), whose rationale for having rules around access to and timing of device use frequently centered around their effects upon their children’s behavior, not in terms of the potential for security breaches, as observed by this mother of two: *“They definitely don’t get them through the week, ever, as you just lose them to the screen and they are like different children afterward*” (Sarah, F, 41 years). Thus, although not a cyber ‘security’ threat as such, the negative effects of technology use on children’s behavior was frequently mentioned by parents as a reason why they limit their children’s access to technology. There was an attitude amongst parents that spending time using technology, even if it was safe and secure, was not necessarily a ‘good’ use of time.

### RQ4: What Are the *Coping Responses* That Families Use in Terms of Strategies to Cope With Cyber-Security Demands?

The strategies used by parents in coping with cyber-security threats and managing cyber-security within the household were clustered into three main sub-themes of *boundaries and rules*, *monitoring*, and *communication and education*.

#### Boundaries and Rules

Many parents (9/14 parents) instigated a set of boundaries and rules around access to devices, timing of use, and which apps children can use or websites they can access. These rules were initially introduced not for security reasons, but with the hopes of minimizing any negative impact upon behavior: “*so when we first got the tablets, they were on it 24/7 and you can notice their behavior changes when they are on it too much?”* (Polly, F, 31 years). Such rules included the days of the week children were allowed to use devices (*“Alfie is banned during the week but he is allowed the iPad or the Fire at the weekend”*: Olive, F, 40 years) and for how long *(“*…*they get them at the weekends for an hour and I put a timer on and they will sit and play games.”*: Sarah, F, 41 years). Parents often also controlled what apps younger children could download on their devices to ensure they were age appropriate, using parental profile settings.

Four parents reported changing their household rules to be more security oriented when their children received a smartphone upon starting secondary school. At this point, rules became focused on protecting children from harm they may encounter online. One way of doing this was to create rules and boundaries around the *type of content* that children could share online, as in the rules being described by this mother of a 12 years old boy: “…*we have rules about not being able to communicate with people he doesn’t actually know*… *if we are on holiday and we post pics we make them ambiguous so no one knows quite where we are or we post them once we are back*.” (Alison, F, 46 years). When using social media, children often described not sharing any identifying content, by using initials instead of names (Thomas, M, 12 years) or not sharing any personal information like phone numbers (*“I’ve never sent someone my number, I only tell someone my number if I’m face to face to them”:* Adam, M, 13 years). Parents also made sure their children were aware it wasn’t safe to communicate with strangers online, whether that was on social media *(“*…*my friend has a public account, and everyone could see her videos and like her music videos. I’ve got a private, I want only my friends to follow me”:* Lottie, F, 12 years) or when doing online gaming (“*When he’s playing Fortnite he can talk to his friends on his headset, but he’s not allowed to talk to anyone else outside of his circle of friends*”: Samantha, F, 49 years, discussing Charlie, M, 16 years). Some children were aware of using passwords to protect their online accounts, as described by this 12 years old boy’s strategy for creating what he felt was a secure password: “*I use a secure password where I have had three previous passwords and I grab bits from all of them and mush them together.”* (Thomas, M, 12 years).

Many parents exerted boundaries around social media, as a way of shielding children from the emotional impact of posting content online, as explained by Polly (F, 31 years) about why she was not allowing her young daughter to use social media: “*Anna (F, 9 years) wants a you tube channel. But its all the negative stuff that goes along with it. People commenting on it and controls you get – they won’t understand why people are being nasty.”* (Polly, F, 31 years). Other parents had rules about which social media platforms their children could access, allowing some but not others. For instance, one 11-year-old girl was permitted to join Instagram and TikTok and to use WhatsApp, but not Facebook due to the age restrictions on Facebook accounts (Tilly, F, 11 years). John (M, 37 years) planned to disallow his children from using social media when they were older (“*I know you are supposed to be over 13 for those, but all the 11 years olds are on those accounts and the parents set it up. And I don’t think they have the emotional ability to cope with it when it goes wrong*.”). Boundaries therefore were designed to shield their children from the worst effects of using technology whether those are behavioral or protecting them from the dangers of interacting with strangers.

#### Battling the Boundaries

Against the background of these boundaries, managing technology use and cyber-security in the family home was often described by parents as a ‘battleground’ (6/14 parents). Children used devices, or banned apps or games, against the express wishes of their parents, as told in this story by a mother of a 7-year old who was playing an online game unbeknownst to his parents: “*We’d try to control what Alfie (M, 7 years) has access to on the iPad so he only has access to what we’ve put on there*…*.but we found that he had access to what’s that game – Fortnite or something? – because his cousin had downloaded it*…*so the two boys had been secretly playing it at Nanny’s house without anyone knowing about it you see.”* (Olive, F, 40 years). Reflecting the importance of peer relationships, children fought against the limits imposed by their parents as to the apps and social media platforms they used. Daisy, mother of a 14 and 7 years old, told a story about her 14 years old son who installed a new messaging app to circumnavigate restrictions on social media: “*I happened to pick up his tablet and words are coming up on his tablet and I’m like ‘that’s odd’, and it was a conversation he was having with somebody via this other media.”* (Daisy, F, 46).

Other children battled against security measures imposed by their parents, as illustrated in this exchange between this mother (Alison, F, 46 years) and their child (Thomas, M, 12 years):

Mother: We use a fake birthdate.
Thomas: Every now and then, yeah.
Mother: We are *supposed* to be using a fake birthdate.

Thus, although parents instilled boundaries, we noticed a tension between parents and children on this issue. Although children did seem to be aware of some of the potential for harm in going online, as earlier illustrated, these concerns did not deter them from breaking their parents’ boundaries and consequently, potentially, leaving the family vulnerable to security threats.

#### Monitoring

Some parents (6/14 parents) managed cyber-security by monitoring their children’s technology use. For younger children this took place physically, in that children were often kept in the same room when using devices. For older children it took the form of virtual surveillance, such as reading their child’s text or instant messages. Sometimes this was done with the explicit consent and knowledge of the child *(“I actually check Leo’s phone and that is the agreement that he has it, and I know the passwords*.”: Jennifer, F, 37 years, speaking about her 13 years old son) and sometimes not – in these cases parents might check their children’s phones, but not necessarily with their explicit knowledge *(“*…*at the moment she doesn’t realize that when she’s asleep I’m reading her phone, I’m going through*.”: Julie, F, 43 years, referring to her 12 years old daughter). Parents also followed their children on social media accounts, so that they could monitor what they were posting online. Parents referred to such strategies as a way of unobtrusively ensuring the physical and emotional safety of their children when interacting online (“*I read just to check everything is above where it should be, to check she isn’t talking to someone she shouldn’t be.”:* Julie, F, 43 years).

#### Communication and Education

Some parents (6/14 parents) managed cyber-security in the family primarily via the relationships with children and educating them as to the potential dangers of going online rather than attempting to control their exposure or shielding them. Some parents combined this approach with other security strategies such as the use of technical solutions (“*I would take that twin thing of trying to have some security settings and also getting them to talk to me*”: Cath, F, 47 years) whilst others acknowledged the futile nature of trying to control what her children were exposed to online, given its growing ubiquity. Rather, they felt that educating and communicating with her children about what they were doing online was a more realistic approach, as described by this mother of two teenage boys: “*However many controls I put in the home, if I limited, if I blocked Wi-Fi, if I limited anything, from what they tell me about what happens at school, they can see whatever they want whenever they want to so for me its more important about making them understand what’s appropriate and what’s not.”* (Samantha, F, 49 years).

### RQ5: What Are Some of the *Social, Technological,* and *Psychological Outcomes* of the Changes in Technology Adoption for Families in the United Kingdom

In terms of the social, technological and psychological outcomes of technology adoption for families, throughout the interviews many parents spontaneously discussed their feelings about the challenges of parenting in the digital age. We generated three themes to define these experiences described by parents. Parents discussed how they live in a ‘*whole new world’*, ushered in by the proliferation of technology into family life, and some parents chose to deliberately *defer or avoid the use of technology* in the home in response to this. Further, some parents described the feeling that the challenges of parenting have not essentially changed, apart from the new digital context: modern parenting is a case of *same same, but different*.

#### A Whole New World

Some parents (6/14 parents) described the feeling that technology is developing and infiltrating family life too quickly for parents to keep up with, sweeping their children’s development along with it: “*It feels like things are happening earlier for our kids then for our generation. I think technology has a lot to do with that*… *for our kids they can talk to their friends in this very private, secrets on their phones and it’s very different*…*they live in a very different world*” (Daisy, F, 46 years). Parents also expressed their feelings that it is out of their control how much their children use technology, and how it is now just ‘the way of the world,’ embedded in all aspects of modern life. Schools were highlighted as an influential institution, both in playing a role in educating children around online safety, but importantly, influencing the age at which children were given their first smartphone. This parent perceived that children at secondary school age were expected to have their own phone, meaning parents have little choice but to ensure their children own one by the age of 11: “*the expectation is by the time they reach secondary school is that they have phones*…*something we’d rather not have started but yes, we don’t seem to have a choice anymore*.” (Rachel, F, 38 years).

#### Deferring or Avoiding Technology in the Home

Parents of younger children sometimes articulated the desire to put the need for cyber-security strategies off until a later stage (4/14 parents). Although only appearing in 4 interviews, this attitude played an important role in these parents’ approach toward cyber-security. These parents described their younger children (between 6 and 10 years old) as being naïve and innocent when it came to the internet, using devices mainly to play games or watch videos, as expressed by this father of a 7 years old boy: “…*to be perfectly honest I don’t think he really knows the internet exists yet, beyond an abstract concept. The iPad for him, it is a mini TV and games console and that is it really*.” (Jack, M, 38 years). As such, these parents expressed relief that they did not need to use any particular strategies to manage cyber security in the home, instead choosing to defer these concerns until their children were older (“*It’s a bit of a you need that bridge when you come to it. Right now we’ve got our own stages to think about, we’re living in a bit where my kids are still so blissfully ignorant.*” (Teresa, F, 42 years, mother of 6 and 8 years old boys).

#### Same Same but Different

This final theme captures instances where parents (8/14 parents) presented the idea that they were living in a ‘new world’ of technology – but that the challenges of parenting are the same. For example, although parents were concerned about online ‘stranger danger,’ it is still the same stranger danger that their own parents had to deal with, as highlighted beautifully by this parent: *“We were told when we were young, “don’t go into the woods because old what’s his name is there” and this is an extension of that, and we are not treating our children any differently, but it’s a much bigger wood*.*”*(Samantha, F, 49 years). One parent told the story of helping their child deal with an instance of chain letters appearing via a messaging app: they explained that chain letters were present in their own childhood, albeit in a different medium. The method of dealing with them had remained the same: “*He said I didn’t want to send it. And I told him you don’t have to, just delete it. I said you don’t need to carry it on nothing is going to happen. They are still going on, just changed how they do it*.” (Polly, F, 31 years, speaking about Jacob, M, 14 years). Similarly, parents felt that their children’s battling against the cyber-security boundaries was just another extension of normal teenage behavior: “*I think it’s a bit like when we were kids, you’d go out and push your time for how long you are out so I think he was pushing to see what he could get away with*.” (Daisy, F, 46 years). In this way, parents in our study expressed their feelings that parenting challenges remain the same as they ever were, but advances in technology mean the context is different.

## Discussion

We firstly discuss how our findings address our five research questions and illustrate ways in which technology adoption in wider society has filtered down to influence cyber-security in the family home, within the framework of the model of social change and human development (Pinquart and Silbereisen, 2004: see Figure 1). Within this theoretical framework, the impact of the introduction of new technologies into society, as a social change, places increased demands on families, both in terms of managing security within the home and the other social and emotional demands of family members using the technologies. We highlight some of these increased demands in terms of the concerns that families have about cyber-security (RQ1). Amongst others, families articulated a range of cyber-security concerns, from online content, online strangers, to financial threats, similar to other research (e.g., Zhang Kennedy et al., 2016). Parental priorities around these threats were underlined by the potential ramifications for the physical and emotional safety of their children, expressing greater concerns around cyberbullying, online stranger danger and online content, compared to financial or technical threats. Families draw on a variety of resources to manage these new demands (RQ2). These resources can be social (such as asking others for help), or personal (such as using their own knowledge to instigate technical solutions) or could be using the security features embedded within the technology itself. However, parents also identified their own perceived limitations in technical knowledge or features of the technology (or media platform) itself as barriers to their ability to cope with the security demands of using technology within the home.

Each family balances the benefits and costs of adopting technology within the home (RQ3), as illustrated in our participants discussions about the positive (enhanced communication within and without the family) and negative aspects (such as perceived detrimental impact upon behavior) of the technology they are using. All these aspects predict how families cope with the demands of adopting new technologies within the home in terms of which strategies they adopt in approaching cyber-security in the family (RQ4). For instance, some families instill rules and boundaries around acceptable online behavior and cyber-risks, and some rely on communication between family members to manage security in the family. We suggest that our final, wider set of themes reflect potential social, personal and technological outcomes (RQ5): families devise new ways of living, because of the rapid development of technology and adoption into family life in terms of new rules and guidelines. Some parents seek to avoid the security and technological implications for as long as possible, and some parents feel the speed in which technology develops means it is difficult to keep their children safe in the digital world.

### How Cyber Security Is Managed in Families

We propose that the variations in coping responses and strategies adopted by parents in managing cyber-security (e.g., monitoring versus communication) likely represents a combination of their perception of acceptable cyber-risk, perceptions about the most effective ways of managing this risk and their parenting style in general. Although we did not explicitly measure or explore parenting styles in this exploratory study, our findings could suggest that parents with a more authoritarian parenting style favor controlling the boundaries of acceptable cyber-security activities and monitoring their children’s online activities as a method of keeping them safe. In contrast, authoritative parents may choose instead to rely on communicating and educating children as to potential dangers online. Supporting this idea, parenting styles have been used to describe how parents mediate their children’s use of media in terms of autonomy-supportive styles (explaining the reasons for rules implemented by parents and considering the child’s perspective), controlling (implementing rules with no say from the child) and inconsistent styles (sometimes rules are implemented, sometimes not: Valkenburg et al., 2013). We suggest general parenting style could thus be a strong influence over approaches to the challenge of managing cyber-security in the family home. Future research should take a more in-depth exploration of parenting style in relation to managing cyber security, to address the question of why particular cyber-security management strategies are preferred by parents over others. Further, an interesting avenue for future research could be to use an assessment of parenting styles in a longitudinal study exploring how various strategies adopted by parents to manage cyber-security relate to developmental outcomes, such as the risky behaviors undertaken by teenagers in terms of cyber security (e.g., Rai et al., 2003).

### Negotiation of Cyber-Security Boundaries

The boundaries of acceptable cyber-security risk within the home, and how these risks were managed were negotiated and re-negotiated as children grew up in the digital world. Initially, boundaries around when, where and how children can use devices were often imposed by parents in order to control the risk of their young children accessing inappropriate content and minimizing the risk of any negative influences upon their behavior. The restrictions around technology use by parents in our study is reflective of the parenting style termed ‘restrictive mediation’ of children’s media use, previously also found to be common in families with young children (e.g., Nikken and Jansz, 2006). Developmentally, as children get older, they become more focused upon their peer relationships (Steinberg, 2005) and new media communication and technologies have now become a significant component of how these relationships are formed and maintained. Thus, boundaries imposed by parents in our study became less focused on access to and time spent on devices, and more around the types of information that can be communicated outside of the family and acceptable levels of risky activities (e.g., acceptable uses of social media platforms and acceptable content to be shared). These boundaries were renegotiated with the introduction of new technologies: for instance, a child may be allowed to use a new form of social media, with the caveat that parents can monitor the posted content.

We noted several cases where parental rules and boundaries around cyber-security in the home was associated with tensions between parents and children, around access to devices, allowable apps, websites and social media platforms, and privacy and security measures used by children. Our findings echo those around tensions in family technology use in other research, reporting that parents trying to restrict device use for their children causes arguments and tensions in the home (Hadlington et al., 2019). Interestingly, parents in our study were cognizant of the fact that these challenges and tensions between parents and children are the same as those of previous generations, but the context is different. As ever, parents are trying to strike a balance between protecting their children, versus allowing them their independence and privacy; between maintaining boundaries for your children versus respecting their need to fit in with their peers. These issues remain for parents these days but there is a new technology-oriented angle, as now as well as wearing all the ‘right’ clothes, kids now need the ‘right’ technology and social media presence in order to fit in with their peers. Further, in line with views from academic research (e.g., boyd, 2014) some parents expressed their understanding that young people, in their desire to maintain near-constant communication with their peers and breaking rules, are not doing anything inherently different to what they always done; it is just that the internet and social media have given them new ways of performing these actions.

Thus, when the issues of managing security in modern family households are considered in the broader social context of technology adoption, these issues are arguably the same as they always have been. The issues of trying to protect children from the dangers of the online world are the same issues that were present prior to the advent of the digital world (e.g., Valentine and Holloway, 2001). Further, it is the parents perceptions of what are the most dangerous aspects of digital living which influence how much time and what their children are allowed to do online, in the same way as parents perceptions of danger outside the house influences how much time children spend outside (Carver et al., 2008). These ideas are echoed in the articulations of the parents in our study, who feel that the fundamental challenges of parenting have not changed, the context has merely evolved.

### Implications, Limitations and Future Directions

As gatekeepers of their children’s use of technology and the internet, all the parents we interviewed had a set of diverse strategies designed to keep their children safe online. However, there is a view that despite such strategies, children are still at risk (Rode, 2009). Our findings are in line with this interpretation and suggest that families are, potentially, doubly exposed to cyber-security threats: sometimes from limitations in parental knowledge due to the rapidly evolving nature of technology, and sometimes from their children pushing the parental boundaries of acceptable cyber-security behaviors. How can we improve this situation? Educating parents and children is one solution, although increasing knowledge, does not necessarily lead to less risky behaviors (Mishna et al., 2009). Given the varying strategies adopted by parents in our study we suggest that a double pronged approach may be useful: empowering parents to educate children in wider, context free safety behaviors and also providing targeted assistance in specific technology based skills (for example, in setting parental filters or privacy settings) where parents feel this would be beneficial. Further, a useful approach may be to encourage parents and children to view family cyber-security as a joint responsibility. Rather than children fighting against their parents’ boundaries, if they are included in setting the boundaries in the first place, this may empower families to navigate the cyber-security world together. Finally, we acknowledge that there will be other local and wider influences upon the cyber-security practices of parents and children that we did not capture. Such influences could include information from the media, peers or the workplace. How these external influences shape cyber-security perceptions and management or could be leveraged to enhance cyber-security within the family remains an avenue for future research.

This was exploratory work and as such provides opportunities for future research to build on our findings as well as address its shortcomings. Firstly, future work should aim to explore cyber security management and negotiation in a larger and more diverse sample of households. Most parents we interviewed were mothers, whereas most children were boys. We acknowledge that this bias in our sample may have influenced our findings and interpretations around perspectives of technology use and preferred cyber-security strategies. Future research in this area should include an equal number of male and female participants, both parents and children, to ensure that any diversity in perspectives related to gender or parental roles is captured. Further, we also recommend conducting interviews across a greater geographical area to capture any differences in local and wider community resources which may impact on parents’ awareness of or implementation of cyber-security strategies. This would also entail a larger sample size compared to our exploratory study, which would help to capture diversity and ensure findings are representative of families in the United Kingdom. Relatedly, in this exploratory study we did not explicitly consider how the age of the child impacts on their cyber-security concerns, management strategies or perceptions of technology. However, we acknowledge that the concerns and perceptions of children can alter as they progress through developmental stages (Steinberg, 2005) and it is possible this applies to cyber-security. Thus, another interesting direction for future work would be to conduct in-depth explorations with children at different ages to see how cyber-security concerns change with age or developmental stages.

We acknowledge that using technology does not always equate to being skilled with technology (Van Deursen and Van Dijk, 2010), and it is likely that digital literacy of parents and children influence how cyber-security is managed within the family. Future studies should thus aim to include objective measures of the level of technological literacy of children and adults alike. Finally, in this exploratory study we did not directly measure possible psychological outcomes of the management of cyber-security in the home (such as feelings of anxiety around technology use, or measures of family functioning or relationship quality) but these present exciting directions for future research.

## Conclusion

In this paper we explored how families in the United Kingdom manage cyber-security within the family home. We provide new knowledge around the demands facing modern families in their management of cyber-security and highlight that the management of cyber security in family homes is an evolving process of negotiation between parents and children. Further, parents in our study expressed the feeling that parenting in the ‘digital age’ is a distinct and new challenge, but at the same time, managing family security is the same challenge it has always been. In this way, they felt that managing cyber-security in the family home is a case of ‘same same, but *different.’*

## Data Availability Statement

The datasets generated for this study will not be made publicly available because the data consists of transcripts of interviews with parents and children and contain personally identifiable information. Requests to access the datasets should be directed to the corresponding author.

## Ethics Statement

The studies involving human participants were reviewed and approved by the Social Science Research Ethics Committee of the University of Bath, United Kingdom (ref S18-013). Written informed consent to participate in this study was provided by the participants’ legal guardian/next of kin. Written informed consent was obtained from the individual(s), and minor(s)’ legal guardian/next of kin, for the publication of any potentially identifiable images or data included in this manuscript.

## Author Contributions

KM contributed to design of the study, conducted and transcribed the interviews, analyzed the data, and contributed to the manuscript writing and revision. AJ contributed to study design and data analysis and provided high level conceptual support.

## Conflict of Interest

The authors declare that the research was conducted in the absence of any commercial or financial relationships that could be construed as a potential conflict of interest.
